# Curriculum renewal in the time of COVID‐19: The Washington University School of Medicine Story

**DOI:** 10.1096/fba.2020-00095

**Published:** 2020-12-22

**Authors:** Eva M. Aagaard, Timothy Yau, Carolyn Dufault

**Affiliations:** ^1^ Division of General Medicine Department of Medicine and Office of Education Washington University School of Medicine St Louis MO USA; ^2^ Division of Nephrology Department of Medicine Washington University School of Medicine St Louis MO USA

**Keywords:** COVID‐19, curriculum renewal, remote learning, technology

## Abstract

Washington University School of Medicine began a curriculum renewal process in 2017 with a goal of implementing the Gateway Curriculum in 2020. In this article, we describe the vision of this curriculum and the infrastructure that was built to support it. We also describe the impact of COVID‐19 on the legacy curriculum and the Gateway Curriculum as well as the lessons learned to date.

## 1 INTRODUCTION

Washington University School of Medicine has been undergoing a comprehensive curriculum renewal over the last 3 years and implemented the first year of the new Gateway Curriculum in the fall of 2020 in the midst of the COVID‐19 pandemic. Much of the infrastructure put in place for curriculum renewal helped to support the sudden transitions required by the COVID‐19 pandemic. Similarly, the lessons learned during the rapid shift to online education have provided valuable information to inform the implementation of the Gateway curriculum. In this article, we describe our vision for the Gateway Curriculum, the infrastructure put into place in support of curriculum renewal, and how that infrastructure supported the rapid shift to online and physically distanced learning brought by the COVID‐19 pandemic. We also describe the lessons learned from these events and how they are shaping the implementation of the new curriculum. We close with our thoughts on how we plan to determine whether these changes will be sustained or transitioned as the COVID‐19 pandemic evolves.

### 1.1 The Vision

Washington University School of Medicine embarked on a curriculum renewal process in the fall of 2017 with a plan to launch the first year of the new Gateway Curriculum in June of 2020.^1^ Through an engaged process involving over 200 faculty, we defined critical priorities for our curriculum renewal that were both consistent with our institutional priorities and based in current and emerging educational evidence. Two key principles in our new curriculum are that it is learner‐centered and sustainable for learners, faculty, staff, and the institution.

Learner‐centered curriculum places the learner and the learning at the center of educational encounters.^2^ To be learner‐centered involves using maximally effective pedagogies including incorporation of technology where it can facilitate learning, using active learning to facilitate deeper knowledge attainment and application of that knowledge, and using facilitated self‐directed learning to inspire lifelong learning and individualized learning plans. Faculty development in pedagogy is critical to the achievement of a learner‐centered curriculum.^3^ Also, technological support, in the form of central resources such as applications, instructional design and videography support, and information technology support are equally critical. Faculty may not possess technologic expertise or may be hesitant to experiment with technology.^4^, ^5^ We hypothesized that by providing at the elbow support in addition to training, adoption may be increased.

To address the sustainability for learners, we focused on the cognitive load of what and how we were teaching.^6^, ^7^ Again, this is facilitated by technology in the form of an effective curriculum and learning management systems that can track workload and learner engagement with material. It also requires effective program evaluation to monitor student perceptions and well‐being. We also focused on the importance of creating programming that promotes the longitudinal relationships with peers and teachers.^8^, ^9^, ^10^ This involves the creation of stable groups of students and highly trained and stable facilitators. In the case of Washington University School of Medicine, this involved the development of a team of coaches trained to facilitate conversations about emotionally charged material but also capable of transmitting assessment feedback and helping learners to develop an individual learning plan to meet necessary competencies and self‐developed career goals. These coaches are provided financial support for protected time to enable their consistent and sustainable engagement. A snapshot of the planned Gateway curriculum was recently published.^1^ Major relevant changes to the planned pre‐clerkship Gateway curriculum from the legacy curriculum included:

1. Transition from a 2‐year pre‐clerkship curriculum to a 16‐month pre‐clerkship curriculum.
2. Transition from a disciple‐based curriculum to one focused on form and function of the human body with consistent integration of basic, clinical, social, and health system science.
3. Use of consistent and predictable active learning strategies including team‐based learning, case‐based learning, and integration of active learning into traditional lectures through a variety of mechanisms. Shortened and animated video recordings were to be developed for content delivery that was required prior to these sessions. Before the COVID‐19 pandemic was fully underway, we had already begun to work with faculty to make a switch from capturing lectures to producing educational videos, all with the support of our newly opened Instructional Design Studio.
4. Integration of clinical skills, community engagement, and coaching throughout the pre‐clerkship curriculum on a weekly basis (initially planned as in person).

### 1.2 The infrastructure build

In order to achieve this vision, critical human and technology infrastructure were needed. Although the Gateway curriculum was not scheduled to begin until fall of 2020, this infrastructure was critical in our response to COVID‐19.

Nearly all technology requires support in the form of people to help it to achieve its intended purpose. The most important early investment in technology at Washington University School of Medicine was purchase of an appropriate curriculum and learning management system to support the Gateway Curriculum. Our legacy systems were poorly integrated, challenging to upkeep and unable to expand to meet our curriculum and assessment mapping needs. Technology integration into our new learning spaces and decisions regarding effective technology supports, including active learning software was also a core necessity. In addition to deciding on these items, we needed a team to build it out, train faculty and staff on their use, and support its effective implementation. An Educational Technology and Innovation Unit (ETIU) was created for this purpose. A new Assistant Dean position was created to oversee the ETIU who has significant experience in both education and technology enhanced learning. A director with extensive educational technology operational experience was hired and three Information Technology (IT) support and programming professionals were engaged to support the required work.

Faculty development in curriculum design and effective pedagogy are foundational to our ability to create and implement the new curriculum. To facilitate this, we created three longitudinal certificate programs under our newly created Academy of Educators. The three programs focus on teaching skills, curriculum design and program evaluation, and learner assessment, respectively. We also delivered a series of faculty development sessions and guest speakers to facilitate critical aspects of the curriculum changes along the way, and repeated these workshops immediately before launch to reinforce the key concepts. Because learning about these ideas is often not enough, we wanted to provide opportunities for basic technical training for our faculty to improve their confidence and support them to make change despite their limited available time. A new Instructional Design Studio was created with a soundproof video‐recording studio, light board and green screen technologies, and a recording booth for supported or independent screencast recording. A fulltime videographer and instructional designer were hired to support faculty actively as they developed their content and programming. This team, along with the ETIU, was also available to all faculty for technical troubleshooting as content was being created.

Finally, in order to evaluate the effectiveness of the new curriculum and the programs and processes we are putting into place, the Program Evaluation and Continuous Quality Improvement Unit (PECQI) was created. Supported by qualitative and quantitative data analysis software and a growing data warehouse, this unit is staffed with an Associate Dean with significant program evaluation and medical education research experience, a manager with education and project management expertise, a data manager, and a data analyst. This process is further supported with a Medical Education Research Unit that includes two senior educational researchers and research support staff.

### 1.3 Enter COVID‐19

In March of 2020, COVID‐19 emerged as a threat to the St Louis region. We identified guiding principles for making decisions about when and how to remove and return learners to the educational environment. Specifically, we made decisions based first and foremost on the safety of our learners, faculty and staff; the ability to achieve learning objectives; and the ability of the system to sustain and support the learning environment. Like nearly all schools in major metropolitan areas, we made a rapid transition to online learning for lectures and other large group activities as soon as community transmission was apparent since these gatherings violated the principle of maintaining the safety of our learners and faculty. A few weeks later, our clinical clerkships were discontinued as the institution made the tough decision to cancel all elective procedures and delay semi‐elective procedures. This resulted in an inability to achieve core learning objectives in two of our required clerkships. In addition, limited personal protective equipment (PPE) suggested the system might not be able to ensure adequate safety. Ultimately, this resulted in removing all learners from the clinical environment.

The ETIU completed a technology readiness survey of our students to determine what kinds of access and management issues our students may face, some of whom chose to leave St Louis and return home. This survey revealed challenges with consistent internet access, competing demands to help care for younger siblings, parents or other family members, and, in some cases, inadequate hardware or software access. This information, combined with the challenges facing our faculty and staff who were also juggling similar challenges, resulted in several key decisions. Sessions that were largely traditional lecture format were changed to asynchronous activities, with resources provided to students to learn at their own pace. In a few instances, new content was rapidly created in the Instructional Design Studio. In other circumstances, prior recordings from previous years were posted. Given the rapid transition, we relied heavily on previously recorded lectures where possible. Small group and some interactive large group activities were primarily transitioned to synchronous Zoom discussions. Another paradigm shift we piloted pertained to the structure and flow of content delivery. With in‐person teaching, many of our lectures were stand‐alone sessions on individual topics, separated in time and space. With asynchronous delivery, we were able to cluster‐related content into organized “sub‐modules” so that students could view all of the related videos and resources on one page in the learning management system. This also allowed us to create formative self‐assessments that were linked to the content and objectives of these modules to enhance self‐directed learning. Synchronous online learning that allowed students to consolidate their learning and apply it in scenarios that are more complex followed this.

For example, in the clinical epidemiology and interpretation of scientific literature content of our traditional doctoring course, content that pertained to study design, fundamentals of measurement (types of data, means, medians, and so on), and principles of diagnostic testing (e.g., sensitivity, specificity, and predictive values) were condensed to multiple 20–30 minute videos and were compiled and embedded on one page for students to review early in the week. An optional multiple‐choice and short answer self‐assessment allowed them to gauge their understanding of the content, and an article that utilized this content in their methods, analysis, and results were provided. Viewing the content of this module and reading of the article were required preparation for a synchronous group discussion at the end of the week. During the group discussion, students analyzed the article, completed exercises and calculations related to the data, and were able to ask questions of an experienced facilitator. A similar format followed for the following weeks, with a new cluster of videos, a new article, and another synchronous remote discussion. All synchronous discussions were recorded, as not every student had reliable internet access all the time. The recordings were posted in the learning management system, which could be viewed later if needed.

There were several lessons learned in our COVID‐19 transitions with regard to use of video‐based education, online synchronous and active learning methods, and faculty engagement. Students preferred the shorter newly recorded videos to those rolled over from prior years. In particular, the audio and video quality of the newer content, and the more condensed nature of these sessions were applauded. This reinforced our need for the Instructional Design Studio and ultimately led to us expanding the staffing in this unit. A small but significant minority of students described struggling to learn the material using videos alone. To support them, we provided PDFs of slides or other materials for note taking and reading. Students voiced a strong need for regular and predictable synchronous sessions to connect with each other and with the faculty. However, synchronous remote teaching also had several challenges. Many of the synchronous activities required thoughtful adaptation to work in a remote environment. We found that discussion was innately more difficult via Zoom than in person and required a decrease in group size when breakout groups were used. However, this increased the number of facilitators needed, which was difficult to accomplish with the rapid shift. The sustainable model of a stable, paid cohort of faculty, as planned for Gateway, would have allowed us to address this more effectively. Lastly, both students and faculty complained of significant Zoom fatigue. Synchronous activities that exceeded 90 minutes were particularly challenging. To address this, we worked to shorten sessions, incorporate breaks, and keep longer sessions interactive through polls and breakout discussion. Courses that involved only a small number of faculty to deliver the majority of its content were more readily able to make these key transitions. Again, this requires effective financial support of key faculty.

### 1.4 Modifications for 2020 to legacy and gateway curricula

#### 1.4.1 Rethinking videos

One of the most significant modifications between the fall and the spring was in our use of recorded videos. In spring 2020, we relied heavily on reuse of existing lecture session recordings from the previous year. This use was only intended as a temporary stopgap measure. In fact, before the pandemic was upon us, we were already creatively rethinking about how, when, and why students use recorded videos in the curriculum. This shift in thinking arose from observations around our students’ reliance on, and preference for, watching recordings of lectures on video rather than attending those lectures in person.

The pandemic accelerated conversations with faculty about shifting the emphasis from simple lecture capture to video production. Drawing on our experience in the transitions and recent evidence around how learners engage with instructional video content, our education team helped to move faculty away from recording 60‐ or 90‐minute videos and toward smaller, more modular video units/chapters that are 10 to 15 minutes.^11^, ^12^ Conversations about what to include in a given video also allowed for a review and recalibration of the alignment between session and course learning goals and the content covered in each video. This was accomplished in part, by asking faculty to develop a script or outline of their session prior to recording in the studio (or from home). A number of faculty were able to substantially reduce the amount of content “covered” and instead focus in depth on a small set of critical ideas aligned with their learning objectives.

Recognizing that a one‐size‐fits‐all approach would not work for producing videos for the Gateway and legacy curriculums, the Instructional Design Studio created a menu of options for faculty for types of videos that they could record at home and/or in the Instructional Design Studio. These options include simple screen recording using Zoom^13^ or more advanced tools that allow for both recording and basic editing. The ID studio also supported and promoted the development of more creative forms of video production, including lightboard recordings, which allows the viewer to see the presenter and the material being presented simultaneously.

As reliance on use of videos increased, we also transitioned from simply posting video files directly into our learning platform, to instead placing all curricular videos in a streaming video player (Kaltura).^14^ Moving to hosting video via a streaming player fits with our learner‐centered approach to the curriculum. Video players like Kaltura are responsive and adapt to different internet speeds and connection bandwidths our students may have, optimizing playback quality across a wide range of conditions. The individual optimization provides best odds for equity in access to video content, which is more important than ever with students scattered across different locations and not always able to access a high‐speed, stable internet connection. Additionally, our new streaming video player allows the student to toggle closed captions on or off, giving them new options around listening or watching the verbal content. Early feedback suggests students value this option.

#### 1.4.2 Integration of Active Learning Approaches in Remote Instruction

At the time of this writing, the fall 2020 Gateway and legacy preclinical curricula are fully underway with first‐year medical students engaged in Gateway and second‐year students participating in the legacy curriculum. Both are largely remote, with about 3 hours per week of the curriculum delivered in person and the other approximately 25 hours of instruction occurring remotely. Clinical learning has resumed largely in person. One of the foundational principles of our new Gateway curriculum is the thoughtful integration of active learning to enhance learner‐centeredness, and we worked to ensure that any remote version of curriculum would still allow us to meet this principle. As described earlier, student feedback from the emergency transition to remote teaching in spring 2020 revealed a clear student need to actively engage in learning and with each other and the faculty in the legacy curriculum as well. To meet these needs for Gateway and legacy curriculum students, we shifted from a primarily asynchronous approach to curriculum delivery to one that emphasized a high degree of flexibility, with plenty of opportunity for synchronous engagement.

For example, using Zoom, we encouraged and supported faculty use of breakout rooms to replicate that intimacy and close personal connection that comes from collaborating and sharing across small groups of peers. For our team‐based learning sessions, which are all delivered remotely, we are still perfecting our Zoom approach, in which faculty facilitators move in and out of breakout rooms checking on each group. This “dropping in” replicates the facilitator role in a classroom setting where they would have moved around the room from group to group. To meet the TBL requirements for group discussion and simultaneous reporting, students will use the collaboration tools within Zoom, including the annotate function, which allows for simultaneous reporting through marking up of documents, slides, or anything else being presented on the screen.

The simplest form of active learning has and continues to be faculty asking questions to prompt individual or group responding. Arguably, in this new remote environment, we have more options than ever to create conversation and discussion. Many of our faculty members have modified how they use Poll Everywhere^15^ (our preferred audience response tool, already in use before COVID‐19 emerged) to deliver interactive polling questions during live Zoom session. For simpler question types, some faculty are using polls that are developed and delivered directly via Zoom. Still others have used the Zoom chat feature as a type of audience response system, asking students to deposit individual comments in the chat to share brief observations, reflections, or questions with the whole class. This has been most effective when a clear plan for who monitors the chat (faculty, teaching assistants, or fellow students) and when students should expect to receive a reply to questions asked via Zoom chat (during the session, at the end of the session, in a FAQ digest after the session) are articulated. An emerging best practice during synchronous Zoom sessions is for faculty to share their ground rules and expectations around engagement and active participation, including communicating preferences for when and how students should ask questions and expectations for muting and unmuting of video and audio.

We are also incorporating opportunities for active learning in videos and other media that students are engaging in asynchronously. During the summer of 2020, we began incorporating interactive quizzes and self‐checks into our produced videos; looking ahead, we plan to add additional forms of interactivity to video and media modules, including branching “choose your own adventure” logic to support practice with clinical reasoning and decision‐making skills.

We see great educational promise in educational experiences that combine both asynchronous and synchronous and active learning elements. This mixed‐methods pedagogical approach shows up frequently in our new Gateway curriculum. For many of the sessions, students complete required prework on their own pace which includes learning new material followed by completing self‐assessment quizzes with immediate feedback. Shortly thereafter, students participate in a synchronous active learning session via Zoom, which requires application of the topics learned during the prework elements, similar to our previously described epidemiology sessions. While this is more work for faculty upfront, it is more sustainable long‐term.

#### 1.4.3 Increased interaction with faculty

Students were very clear about their need for an engaged, aware, and supportive faculty presence for all aspects of the curriculum, including––and especially––for those elements being delivered remotely. To assure this happens, in our Gateway and legacy curriculums we are ensuring we have a thoughtful and appropriate mix of asynchronous and synchronous remote sessions, along with in‐person sessions where it is safe to do so and the learning objectives require in‐person interaction. Examples of this latter category include clinical skills practice, coaching groups, and small group sessions that focus on sensitive or personal discussion topics. We have found that these in‐person sessions provide much needed engagement for both students and faculty, ultimately contributing to the sustainability for both groups during this challenging time.

As we start out in the fall of 2020, the majority of our sessions within our Gateway and legacy courses are offered synchronously via Zoom, with students having the option to participate in real‐time or watch the session recording at a time of their choosing asynchronously. In addition, a new request has been made from the students to have an asynchronous question and answer platform such that interactions with faculty and with each other around content can be expanded. We are actively monitoring the sustainability of this request on faculty time.

#### 1.4.4 Use of feedback to inform rapid and deliberate change

We have relied on multiple sources of feedback to inform the curriculum modifications described here, and given our changed world, there are also planned modifications to when and how we will collect feedback. We will continue to collect student feedback on teaching and learning experiences through the typical channels, including surveys and advisory groups. In addition, for the Gateway curriculum, we have created regular focus groups to assess in real‐time what is and what is not working in the curriculum and to allow the students to suggest modifications as we go.

However, we now also have a rich world of data analytics from our teaching platforms that provide, among other things, granular data on student engagement with session resources attached to our event pages. For example, we can see, to a person, which students have and have not opened or viewed the required and optional resources provided on each session page. Students are aware that this information is collected and in their view of the platform, they see a green checkmark indicating that they have seen or opened a given resource.

Through our streaming video player, we also have access to the aggregated data of video engagement patterns. With this information it is easy to observe patterns of student use and engagement, including identifying sections of a video that were not viewed at all and areas watched multiple times. Returned to the faculty, this type of information contributes to the quality improvement process by providing objective data about how students are using or not using the video resources faculty take great care and time to create.

#### 1.4.5 Communications modifications

We recognize that making all of these critical modifications alone would not work without a robust communications plan for all stakeholders, including students, faculty, and our administrative staff. As an example, with the new variety of session types in the curriculum, we needed a consistent naming approach to classify our sessions that would be consistent from course to course. All session titles in our learning management system are preceded with short codes indicating if they are asynchronous, synchronous, or in person; whether attendance is required and tracked; and whether there is required preparation. All required preparation elements include time estimates to help students plan and allocate effort during the week.

We have also found we cannot over communicate with our students. In our new normal, course faculty now always include opportunities for synchronous office hours or review sessions. Likewise, in our Gateway curriculum, course communications have been standardized to include a weekly “look ahead” email from the course leaders, ensuring students are alerted to the key events, tasks, and requirements. This weekly round‐up approach builds on past success in the legacy curriculum, in which some courses took this approach.

One of the most important communication tools we have is consistency and templatization of how we build our courses and events. We were fully prepared to shift to remote learning in March of 2020 even though we had a nearly complete in‐person pre‐clerkship curriculum. The success of our transition was based in part on our courses and sessions having a consistent, structured digital presence. Because we did not need to build these digital course homes during the emergency transition phase, we were able to easily communicate––via each session page––clear information on how the session had been transitioned for remote learning. To support the continued improvements to our structured and consistent organization in our learning management system, we developed new collector tools to help faculty to transmit information about sessions, courses, and assessments. This systematic collection allows us to enter the information into our teaching and learning platform with the clarity and consistency our students have now come to expect. While there is some natural tension in balancing faculty autonomy in the design of the session and course pages, we see standardization as necessary to fulfill our promise to deliver a curriculum that is, at its core, learner‐centered.

### 1.5 Conclusions

Reflecting on the last 6 months and looking to the future, a few things are clear. The COVID‐19 pandemic forced rapid implementation of some plans that were already in process, while also pushing innovation in areas of the curriculum that were relatively untouched. The faculty, staff, and students were prepared for innovation because of the curriculum renewal process and we had fortunately put much infrastructure into place that helped to facilitate the necessary changes. Nevertheless, the entire process has been grueling for all involved. In addition to the emotional toll of the pandemic and racial injustice of 2020, our students, staff, and faculty have been in a constant state of change for not only the implementation of the Gateway Curriculum, but also the mass revision of the legacy curriculum. Our PECQI team and the medical education research unit have put into place plans to evaluate the changes made in both the Gateway and legacy curricula. We will use this information to guide our future plans in an effort for continuous improvement. Just as a learner‐centered approach promotes self‐directed growth for the individual, this data will allow our curriculum and institution to continually improve as well.

## CONFLICT OF INTEREST

The authors report no conflict of interest.

## AUTHOR CONTRIBUTIONS

All three authors made substantive contributions to the writing and editing of the manuscript.
